# Hyperglycemia Increases Muscle Blood Flow and Alters Endothelial Function in Adolescents with Type 1 Diabetes

**DOI:** 10.1155/2012/170380

**Published:** 2012-06-03

**Authors:** Amanda S. Dye, Hong Huang, John A. Bauer, Robert P. Hoffman

**Affiliations:** ^1^Department of Pediatrics, West Virginia University, Charleston, WV 25302, USA; ^2^The Research Institute at Nationwide Childrens Hospital, Columbus, OH 43205, USA; ^3^Division of Pediatric Endocrinology, Metabolism, and Diabetes, Department of Pediatrics, and the Clinical Research Center, The Ohio State University College of Medicine and Public Health, Columbus, OH 43205, USA

## Abstract

Alterations of blood flow and endothelial function precede development of complications in type 1 diabetes. The effects of hyperglycemia on vascular function in early type 1 diabetes are poorly understood. To investigate the effect of hyperglycemia on forearm vascular resistance (FVR) and endothelial function in adolescents with type 1 diabetes, FVR was measured before and after 5 minutes of upper arm arterial occlusion using venous occlusion plethysmography in (1) fasted state, (2) euglycemic state (~90 mg/dL; using 40 mU/m^2^/min insulin infusion), and (3) hyperglycemic state (~200 mg/dL) in 11 adolescents with type 1 diabetes. Endothelial function was assessed by the change in FVR following occlusion. Seven subjects returned for a repeat study with hyperglycemia replaced by euglycemia. Preocclusion FVR decreased from euglycemia to hyperglycemia (*P* = 0.003). Postocclusion fall in FVR during hyperglycemia was less than during euglycemia (*P* = 0.002). These findings were not reproduced when hyperglycemia was replaced with a second euglycemia. These results demonstrate that acute hyperglycemia causes vasodilation and alters endothelial function in adolescents with type 1 diabetes. In addition they have implications for future studies of endothelial function in type 1 diabetes and provide insight into the etiology of macrovascular and microvascular complications of type 1 diabetes.

## 1. Introduction

Multiple studies have indicated that cardiovascular disease has its origins in childhood and adolescence [1–3]. This is particularly true in individuals with type 1 diabetes [4, 5] and is of concern since vascular complications are the primary causes of morbidity and mortality in individuals with both type 1 and type 2 diabetes. Functional abnormalities of the vascular endothelial lining have been shown to precede overt cardiovascular disease in patients with and without diabetes [1, 2, 6, 7]. Furthermore, abundant short-term evidence indicates that endothelial dysfunction precedes microvascular, as well as macrovascular complications in patients with type 1 diabetes [8–10].

Previous studies have indicated that endothelial dysfunction is present in adolescents with type 1 diabetes before the development of overt complications [4, 5, 11, 12]. However, these studies have assessed endothelial function in subjects with type 1 diabetes in the fasting condition with wide variations in glucose levels. Acute hyperglycemia, however, has been shown to increase muscle blood flow [13] and decrease endothelial function in normal adults [14]. Acute reduction of hyperglycemia in type 1 diabetes partially restores but does not normalize endothelial function [15]. The acute effects of increases in plasma glucose on muscle blood flow and endothelial function in adult or adolescent subjects with type 1 diabetes have not been studied. Therefore, the primary objective of this study was to determine how acute changes in plasma glucose alter vascular function in adolescents with type 1 diabetes.

We measured forearm blood flow and endothelial function in otherwise healthy, pubertal subjects with type 1 diabetes at variable fasting glucose levels, and during euglycemic and hyperglycemic insulin infusion. Secondarily, we assessed other cardiovascular risk factors including inflammatory markers and markers of oxidative stress in all three conditions.

## 2. Methods

### 2.1. Subjects

Eleven adolescents (4 female, 7 male) with type 1 DM were recruited from the Pediatric Diabetes Clinic of Nationwide Children's Hospital (NCH). Their mean age was 14.5 ± 1.0 years (mean ± SD) and their mean body mass index was 21.5 ± 2.9 kg/m^2^. Mean HgbA1c of 8.3 ± 1.2% and mean duration of diabetes was 4.2 ± 3.9 years. The study was approved by the NCH Institutional Review Board and informed consent was obtained from a parent or legal guardian. Proper assent was obtained from all subjects.

Screening included a history, physical exam, Tanner staging, and fasting laboratory testing. Type 1 DM was defined by American Diabetes Association Criteria plus a fasting C-peptide of less than 0.4 ng/mL, insulin monotherapy since diagnosis, and an absence of a history of oral hypoglycemic agents and acanthosis nigricans on exam. All subjects were nonsmokers by report.

All subjects were Tanner stage 2–4 in order to minimize the effects of starting or finishing puberty. In order to limit confounding effects on endothelial function, subjects with BP > 95th percentile, smoking, pregnancy, and uncorrected hypothyroidism, were excluded. Subjects with microalbuminuria, overt nephropathy, or early renal failure (random urine microalbumin/creatinine > 0.02 mg albumin/mg creatinine; serum creatinine > 1.0 mg/dL) were also excluded.

### 2.2. Protocol

For the main study visit, subjects were admitted to the Clinical Research Center (CRC) at Ohio State University after an overnight fast. Subjects continued their insulin regimen of multiple dose injections (MDI) or continuous subcutaneous insulin infusion (CSII). Subjects on MDI received basal insulin injection the night prior to study and subjects on CSII continued with basal insulin infusion overnight. Subjects were NPO with subsequent omission of morning insulin bolus on the study day. Endothelial function measurements and blood sampling for laboratory analysis of inflammatory, oxidative, and endothelial markers were performed in each subject during three states: (1) fasting, (2) euglycemia, and (3) hyperglycemia.

#### 2.2.1. Assessment of Endothelial Function

Forearm Blood Flow (FBF) was measured using strain gauge venous occlusion plethysmography, as previously described by Higashi and Yoshizumi [16], using a Hokanson EC6 plethysmograph (DE Hokanson Inc, Bellevue, WA) in the dominant arm. An indium-in-silastic strain gauge was attached to the widest portion of the forearm and connected to a plethsmography device. Sphygmomanometric cuffs were placed on the arm at the wrist and on the upper arm. The wrist cuff was inflated to 200 mmHg to occlude blood flow to the hand for the duration of the study. During FBF measurement, the upper arm cuff was inflated to 40 mmHg for 10 out of 15 seconds to occlude venous return but not arterial inflow Each subject had two minutes of baseline flow recorded and then the upper arm cuff was inflated to 200 mmHg pressure for five minutes to occlude arterial flow to the arm. It was then released to create a sudden shear stress. FBF was again measured for the next minute. The FBF outflow signal was transmitted to a recorder (Powerlab 8, ADInstruments, Colorado Springs, CO) and FBF was expressed as mL per minute per 100 mL of forearm tissue volume. Forearm vascular resistance (FVR) was determined by mean arterial pressure (MAP, measured by automated sphygmomanometer) divided by FBF. All studies were scored by a single experienced investigator (RPH). The reactive hyperemic change in FVR from before to after occlusion was used to measure endothelial function.

#### 2.2.2. Insulin Clamps

**Hyperglycemic Study**

After the fasting endothelial function measurement was complete, the insulin clamp portion of the study was initiated. A catheter was placed in an antecubital vein for the administration of glucose, insulin, and saline. Insulin infusion was initiated at a rate of 40 mU/m^2^/min to bring the glucose level to a target of 90–95 mg/dL. Insulin rates were increased above 40 mU/m^2^/mn in 2 subjects because of prolonged hyperglycemia. Blood samples were taken through a second intravenous catheter at five-minute intervals for the immediate determination of plasma glucose using an automated glucose oxidase technique (YSI Model 2300; Yellow Springs, Instruments, Yellow Springs, OH). When the target glucose level was achieved, dextrose was added to maintain euglycemia for 30 minutes with a minimum total of 60 minutes of insulin infusion. In those subjects in whom the insulin level was increased, it was reduced to and maintained at 40 mU/m^2^/min at the beginning of the euglycemic period. Endothelial function measurement and blood sampling were repeated at the end of 30 min of euglycemia.

After completion of the euglycemic phase the insulin infusion was continued and the dextrose infusion rate was increased to raise the plasma glucose level to a target of 200 mg/dL for 60 minutes following the end of euglycemia. Endothelial function and biochemical markers were again measured after hyperglycemia.

**Euglycemia Control Clamp**

As a control for the effects of fluid volume and insulin infusion, seven subjects returned for an additional study in which the hyperglycemic arm of the study was replaced with a second euglycemic phase. Each subject received normal saline to maintain an equal rate of volume infusion as in the previous hyperglycemic clamp. The insulin infusion rate remained unchanged.

#### 2.2.3. Laboratory Measurements

High-sensitivity C-reactive protein (hsCRP), total plasma antioxidant capacity (TAOC), and a measure of oxidative stress, were measured for each subject at each stage of glycemia. TAOC is a nonspecific assay of antioxidant defense which measures the ability of constituents in plasma to absorb oxidation (BioVision Research Products, Mountain View, CA). Soluble intracellular adhesion molecules (sICAM) were also measured at each stage of glycemia as a marker of endothelial activation. Serum sICAM levels were determined using a commercially available assay (R & D Systems, Minneapolis, MN; Cat # BBE 1B).

#### 2.2.4. Statistical Analysis

Repeated measures analysis of variance was used to determine differences in FBF and FVR responses to changes in plasma glucose and upper arm occlusion. Only glucose was used as a repeat factor for TAOC, sICAM, and hsCRP. Systat 11 (SAS, Systat Software Inc, Chicago, IL) was used to perform all statistical analysis. Data are expressed as mean ± SE. Differences were considered significant at *P* < 0.05 and tendencies are mentioned at *P* < 0.1.

## 3. Results

### 3.1. Hyperglycemic Study

**Figure 1:**
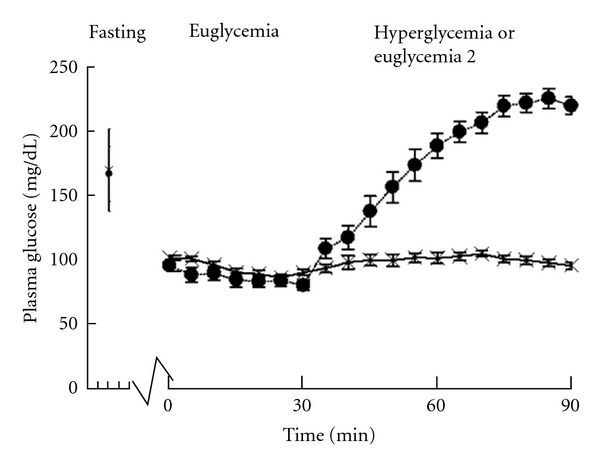
Serum glucose values during hyperinsulinemic hyperglycemia insulin clamp (solid circle) and the euglycemia control clamp (X). Fasting glucose values are indicated by the vertical line. The average time to achieve euglycemia was 57 ± 11 minutes during the hyperglycemia clamp and 70 ± 17 minutes during the euglycemia control clamp. During euglycemia study, normal saline was given to match volume of dextrose 20% given during hyperglycemia study.

During the first study clamp, the mean fasting glucose was 166 ± 21 mg/dL, mean glucose during euglycemia was 83 ± 4 mg/dL, and mean glucose at the end of hyperglycemia was 219 ± 7 mg/dL (Figure 1). The mean duration to achieve euglycemia was  57 ± 10  minutes. 

**Figure 2:**
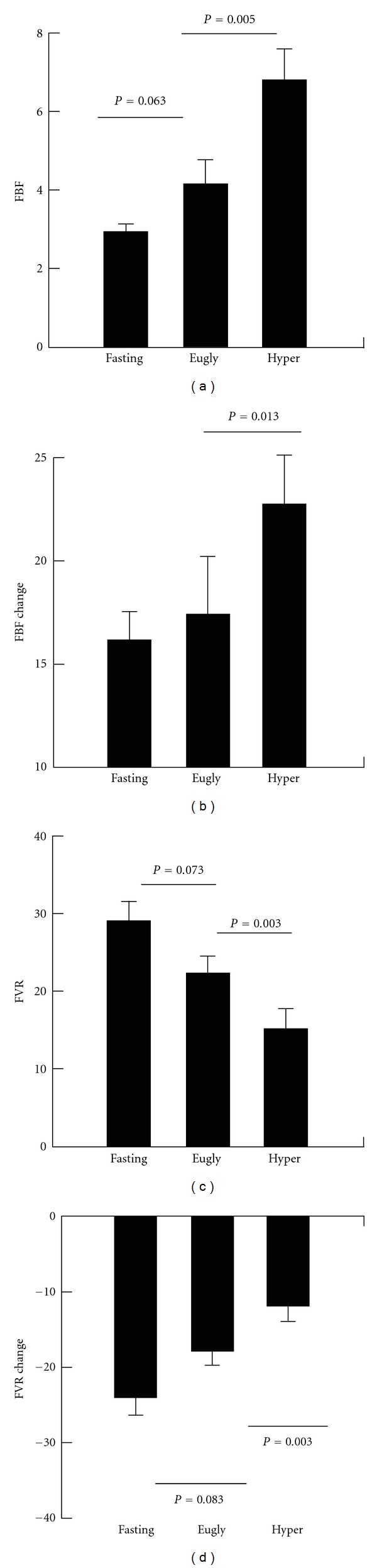
Preocclusion forearm blood flow (FBF, (a)), forearm vascular resistance (FVR) (c) and changes in FBF and FVR from pre- to postocclusion (b,d) fasting and during hyperinsulinemic clamp with euglycemia followed by hyperglycemia. FBF measured in mL per minute per 100 mL of forearm tissue volume; FVR determined by MAP (mean arterial pressure)/FBF. Eugly : euglycemic period, Hyper : hyperglycemic period. Lines indicate between group differences.

Repeated measures analysis of variance revealed significant differences in FBF across the three glucose levels (fasting, euglycemia, and hyperglycemia; *P* < 0.001) and a significant difference in response to upper arm occlusion across the three levels (*P* = 0.002, Figures 2(a) and 2(b)). Specifically, preocclusion FBF tended to increase from fasting to euglycemia (*P* = 0.063) and further increased from euglycemia to hyperglycemia (*P* = 0.005). The increased FBF following upper arm occlusion during hyperglycemia was greater than the increase following occlusion during euglycemia (*P* = 0.013). Repeated measures analysis revealed a tendency for a decrease in the ratio of postocclusion FBF to preocclusion FBF across the three levels (*P* = 0.070). Although no specific differences were noted between baseline and euglycemia or between euglycemia and hyperglycemia. 

During the hyperglycemia clamp, an increase in systolic blood pressure was noted (*P* < 0.001) across the three phases of the clamp (Table 1). There were no differences identified during the hyperglycemic clamp for diastolic blood pressure or mean arterial pressure. For FVR (Figures 2(c) and 2(d)) repeated measures analysis of variance again demonstrated a significant effect of glucose level on FVR (*P* < 0.001) and a significant effect of glucose level on the occlusion-induced change in FVR (*P* = 0.001). Specifically, preocclusion FVR tended to decrease from fasting to euglycemia (*P* = 0.073) and significantly decreased from euglycemia to hyperglycemia (*P* = 0.003). The absolute postocclusion decrease in FVR during euglycemia tended to be less than that at baseline (*P* = 0.083) and the postocclusion fall in FVR during hyperglycemia was significantly decreased compared to euglycemia (*P* = 0.003). However, no differences were seen for the pre- to postocclusion percent decrease in FVR between the three stages (Table 1).

Significant decreases in sICAM values across all three phases were present (*P* = 0.002). The most significant difference was identified between fasting and euglycemia (*P* = 0.058) concurrent with insulin initiation. No significant changes were identified between the three glycemic states for TAOC or hsCRP.

### 3.2. Euglycemia Control Study

To assure that changes seen during hyperglycemia in the previous study were not due to time, continued insulin infusion or volume infusion, seven subjects returned for repeat studies. Baseline and euglycemia were identical to previous study after which euglycemia was maintained and normal saline was given at an identical rate to the dextrose infusion in the hyperglycemia study over the last hour. Mean fasting glucose was 169 ± 32 mg/dL, mean glucose during euglycemia phase 1 was 89.2 ± 2.2 mg/dL, and mean glucose during euglycemia phase 2 was 94.6 ± 2.9 mg/dL. The mean duration to euglycemia was  70 ± 17 minutes. This was not different from the hyperglycemic clamp.

**Figure 3:**
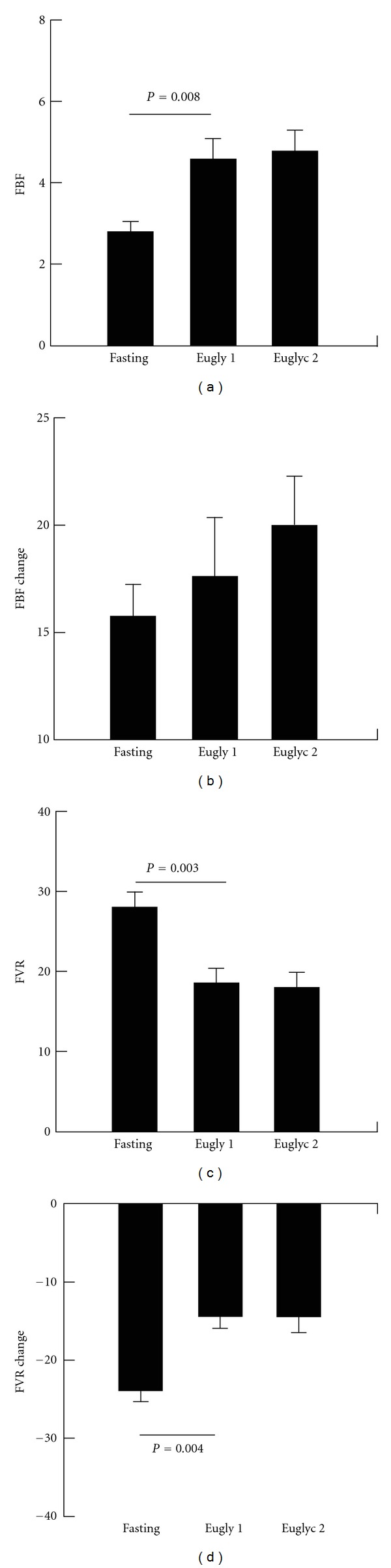
Preocclusion forearm blood flow (FBF, (a)), forearm vascular resistance (FVR) (c) and changes in FBF and FVR from pre- to post-occlusion (b,d) fasting and during hyperinsulinemic clamp with euglycemia periods 1 and 2. FBF measured in mL per minute per 100 mL of forearm tissue volume; FVR determined by MAP (mean arterial pressure)/FBF. Eugly 1 : first euglycemic period, Eugly 2 : second euglycemic period. Lines indicate between group differences.

Repeated measures analysis of variance again revealed significant differences in FBF across the three measurement times (baseline, euglycemia 1, and euglycemia 2; *P* = 0.026, Figures 3(a) and 3(b)) Preocclusion FBF increased from baseline to euglycemia (*P* = 0.008) but did not increase further during the second euglycemic period. There were no differences in response to upper arm occlusion between the three time periods. The increase in preocclusion FBF from euglycemia to hyperglycemia during the first study tended to be greater than the lack of change from the first to second euglycemic periods (*P* = 0.082).

Similar to the hyperglycemic clamp, there was an increase in systolic blood pressure noted as the clamp study proceeded (*P* = 0.003) but no differences were noted in regards to diastolic blood pressure or mean arterial pressure (Table 1). For FVR repeated measures analysis of variance, again, demonstrated differences in FVR across the three study periods (*P* = 0.002, Figures 3(c) and 3(d)) and significant differences in response to upper arm occlusion between the glucose levels (*P* = 0.002). As in the hyperglycemic study preocclusion FVR fell from baseline to euglycemia (*P* = 0.003) but did not differ between the two euglycemic periods. Similarly, the decrease in FVR following upper arm occlusion was less during first euglycemic period than at baseline (*P* = 0.004) but did not differ between the two euglycemic periods. In regards to occlusion response, the percent change in FVR during the first euglycemic period tended to be smaller than that at baseline (*P* = 0.067, Table 1).

TAOC and hsCRP decreased from fasting values during the clamp (Table 1).

**Figure 4:**
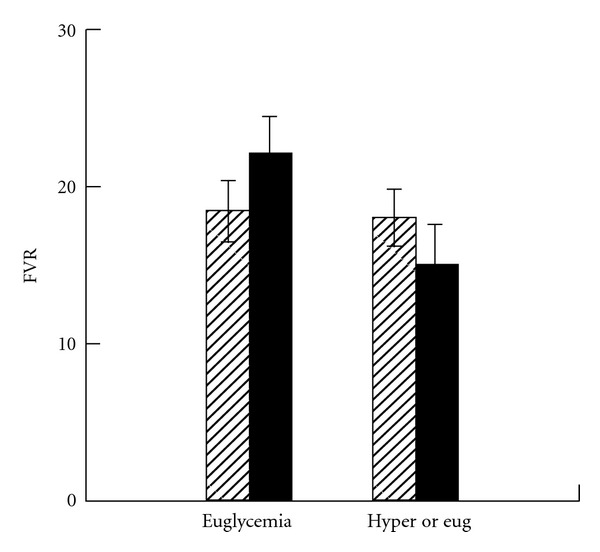
Forearm vascular resistance (FVR) during common euglycemic period followed by either hyperglycemia (solid bars) or second euglycemia period (open bars) with continuous insulin infusion throughout. During euglycemia study, normal saline was given during second euglycemic period to match volume of dextrose 20% given during hyperglycemia study. The fall in FVR during hyperlycemia was significantly different from the lack of change during euglycemia (*P* = 0.042).

Comparison of the changes between euglycemia and hyperglycemia with the changes between first and second euglycemia periods revealed that the increase in preocclusion FBF from euglycemia to hyperglycemia was significantly different from the lack of change during continued euglycemia (*P* = 0.049). There were no significant differences found between the studies for postocclusion FBF response. For FVR, the decrease in preocclusion FVR from euglycemia to hyperglycemia was significantly different from the lack of change during continued euglycemia (Figure 4, *P* = 0.042) and the decrease in postocclusion fall in FVR during hyperglycemia was significantly different from the lack of change during continued euglycemia (*P* = 0.047).

## 4. Discussion

Hyperglycemia causes acute vasodilation in healthy adults [13]. The vasodilation is due to osmotic effects of hyperglycemia since similar changes in FVR occur during mannitol infusion but not during 0.2% saline. The current study indicates that acute hyperglycemia has similar effects in adolescents with type 1 diabetes. The vasodilatory effect of hyperglycemia was demonstrated both by an increase in FBF and a decrease in FVR. The changes in both during hyperglycemia were different from the lack of increase during similar volume infusion (0.9% saline) with maintenance of euglycemia and thus cannot be attributed to volume or continued insulin infusion.

The mechanism for hyperglycemia-induced vasodilation is not clear. One possible mechanism would be increased vascular volume and baroreflex suppression since systolic blood pressure increased during hyperglycemia. Against this hypothesis is that a similar, although not statistically significant, increase occurred during the same time period of the control, euglycemic saline infusion study without changes in FBF or FVR. Also, against this hypothesis is the previous study which showed that hyperglycemia increases, not decreases, sympathetic nerve activity [13]. Since hyperglycemia increases reactive oxygen species which decrease nitric oxide availability [2, 6], it is highly unlikely that hyperglycemic vasodilation is endothelially mediated. Thus, further study will be necessary to investigate potential mechanisms.

In contrast to the acute vasodilatory effect of hyperglycemia during constant insulin infusion, we also saw vasodilation with correction of hyperglycemia from baseline to euglycemia during both studies. This is likely due to the vasodilator effect of insulin infusion [17]. This demonstrates the importance of studying changes in glucose without changes in insulin.

The direct impact of hyperglycemia-induced vasodilation in patients with type 1 diabetes is not certain. One primary area of interest would be its effect on diabetes complications, nephropathy in particular. Specifically, hyperglycemia-mediated decreased vascular resistance may be responsible for increased renal blood flow and increased glomerular filtration rate seen early in diabetic nephropathy [18]. Additional evidence that hyperglycemia-induced vasodilation plays a role in microvascular complications comes from the fact that hyperglycemia increases retinal blood flow in type 2 diabetes [19] and that increased retinal blood flow has been associated with more rapid progression of diabetic retinopathy [20]. In adolescents with type 1 diabetes, hyperglycemia is frequently present postprandially and acute vasodilation caused by these recurrent hyperglycemic episodes may play a long-term role in the microvascular damage that occurs in patients with type 1 diabetes. It is, thus, important to assure that appropriate rapid acting insulin is given before each meal to decrease hyperglycemia induced vascular dysfunction.

The effect of hyperglycemia on endothelial function in our study is unclear. The total postocclusion fall in FVR was less during hyperglycemia but the percent fall in FVR was not different. The smaller absolute postocclusion vasodilatory response during hyperglycemia is most likely due to the increased preocclusion vasodilation and decreased reserve capacity for additional stress-induced vasodilation or, in other words, a ceiling effect in maximal vasodilation. Kawano et al. [14] previously reported that hyperglycemia decreases brachial artery flow-mediated vasodilation during hyperglycemia in healthy adults and subjects with impaired glucose tolerance and type 2 diabetes. Chittari et al. [19] confirmed these findings in adults with type 2 diabetes. Neither study reports whether the decrease was due to pre- or postocclusion differences. The current study adds to these two studies in two ways. First, they assessed the flow-mediated increase in brachial artery diameter, a conduit vessel, while the current study measured FBF and FVR and assessed resistance vessel function. Second, since both of these studies used oral glucose tolerance testing to induce hyperglycemia, insulin levels increased at the same time as plasma glucose levels. In the current study hyperglycemia was induced following euglycemic insulin clamp with continued insulin infusion in insulin deficient type 1 diabetes so that insulin levels should not have changed. Thus, the effects seen are clearly due to hyperglycemia and not hyperinsulinemia. In vitro studies, in isolated rat mesenteric arteries confirm that hyperglycemia, directly, impairs the vasodilatory response to acetylcholine [21]. This finding also indicates that diminution of endothelial function by hyperglycemia extends beyond simply increasing baseline flow.

Multiple past studies have evaluated endothelial function in adolescents with type 1 diabetes [4, 5, 11, 12]. These studies have consistently demonstrated impaired endothelial function in subjects with type 1 diabetes compared to control subjects [4, 5, 12]. Unfortunately, since these studies did not control for varying fasting blood glucose levels, the fact that hyperglycemia acutely alters postocclusion responses raises concerns regarding the interpretation of these findings. Future studies comparing endothelial function between subjects with diabetes and controls will need to account for differences in plasma glucose levels. Unfortunately, our results also indicate that this cannot be done simply by acute infusion of insulin since preocclusion FBF was increased and FVR decreased during the euglycemia compared to fasting. This is likely due to the well-established vasodilatory properties of insulin [17]. This led to a trend toward a decreased reactive hyperemic response during simple euglycemia.

Limitations to this study are the short duration of hyperglycemia and the study of males and females combined. The lack of effects of hyperglycemia on markers of endothelial damage, inflammation or oxidation, may be secondary to the short duration of hyperglycemia in our study. It is possible that more prolonged hyperglycemia may have induced changes in some of these areas. Although gender differences have been found in endothelial function in adults, no differences were apparent in this study.

In conclusion, acute hyperglycemia has profound effects on blood flow, vascular resistance, and endothelial function. These findings have implications for future studies of endothelial function in type 1 diabetes. More importantly, hyperglycemic-induced vasodilation may play a significant role in the development of macrovascular and microvascular complications in patients with type 1 diabetes.

##  Authors' Contribution

A. S. Dye participated in data collection and writing paper. H. Huang performed laboratory measurement. J. A. Bauer supervised laboratory measurements and reviewed edited paper, R. P. Hoffman wrote protocol and obtained research funding, supervised or directly collected data, wrote and edited paper. He is responsible for its content.

## Figures and Tables

**Table 1:** Blood pressure, EPC, inflammatory and oxidative measures during hyperglycemic clamp and euglycemic control clamp. All values are expressed as mean ± standard error.

	Hyperglycemia clamp (*n* = 9)			*Overall P* * value*	Euglycemia control clamp (*n* = 7)			* Overall P* * value*
	*Fasting*	*Euglycemia*	*Hyperglycemia*		*Fasting*	*Euglycemia 1*	*Euglycemia 2*	
Systolic blood pressure (mmHg)	105 ± 2	107 ± 2	113 ± 2*	<0.001	102 ± 2	107 ± 2*	113 ± 4	0.003
Diastolic blood pressure (mmHg)	61 ± 3	63 ± 3	61 ± 2	NS	57 ± 4	58 ± 2	61 ± 3	NS
Mean arterial pressure (mmHg)	75 ± 2	77 ± 2	79 ± 2	NS	72 ± 2	74 ± 2	78 ± 2	NS
Reactive hyperemia (% change in FVR)	−82.1 ± 1.3	−80 ± 1.6	−77.7 ± 2.6	NS	−84.1 ± 1.2	−77.9 ± 2.4^#^	−79.6 ± 2.5	0.083
Antioxidant capacity (mM Trolox equivalent)	108 ± 8	106 ± 7	98 ± 6	NS	129 ± 2	126 ± 2	118 ± 2^∗†^	0.001
sICAM (ng/mL)	162 ± 7	156 ± 8	151 ± 7	0.002	155 ± 10	151 ± 13	146 ± 11	NS
hsCRP (mg/L)	0.95 ± 0.65	0.98 ± 0.67	0.82 ± 0.60	0.057	0.75 ± 0.27	0.68 ± 0.24	0.68 ± 0.26^†^	0.049

*P < 0.05 versus previous study phase, ^#^ *P* < 0.1 versus fasting, ^†^ *P* < 0.05 versus fasting.
